# Association between Chronic Laryngitis and Particulate Matter Based on the Korea National Health and Nutrition Examination Survey 2008–2012

**DOI:** 10.1371/journal.pone.0133180

**Published:** 2015-07-15

**Authors:** Young-Hoon Joo, Seong-Soo Lee, Kyung-do Han, Kyung-Ho Park

**Affiliations:** 1 Department of Otolaryngology-Head and Neck Surgery, College of Medicine, The Catholic University of Korea, Seoul, Korea; 2 Department of Endocrinology, College of Medicine, The Catholic University of Korea, Seoul, Korea; 3 Department of Biostatistics, College of Medicine, The Catholic University of Korea, Seoul, Korea; Utah State University, UNITED STATES

## Abstract

**Background:**

Chronic laryngitis (CL) has been described as chronic inflammation of the larynx. CL have various causes such as long-term smoking, acid reflux, voice overuse, bronchitis, allergies, pneumonia, excessive exposure to toxic chemicals and complications from the flu or a chronic cold. However, the prevalence of CL and role of air pollution in the etiology is uncertain.

**Objective:**

The aim of this study was to investigate the relationship between CL and particulate matter with aerodynamic diameter less than 10 μm (PM_10_) in South Korea using data from the Korea National Health and Nutrition Examination Surveys (KNHANES) during 2008–2012.

**Methods:**

KNHANES is a cross-sectional survey of the civilian, non-institutionalized population of South Korea (n = 21,116). A field survey team that included an otolaryngologist moved with a mobile examination unit and performed interviews and physical examinations. The mean annual concentrations of ambient PM10, SO2, O3, NO2, and CO levels in Korea were determined from monitoring station data. Multiple logistic regression was used to examine the relationship of air pollution to CL.

**Results:**

Among the population ≥ 19 years of age, the weighted prevalence of CL was 3.37 ± 0.30% (95% confidence interval, 2.79–3.95%). CL was more prevalent in men, current smokers, and those with lower household income and prevalence increased with age. A significant decrease over time was observed in the prevalence of CL (P for trend = 0.0049) and the annual average concentrations of PM_10_ (P for trend < 0.0001) from 2008 to 2012. In a multivariate model, the factors associated with CL included PM_10_ (odds ratio [OR], 1.378, p = 0.0457), age (OR, 1.020, p<0.0001), sex (OR, 0.734, p = 0.0179), and smoking status (OR, 1.438, p = 0.0054).

**Conclusion:**

Elevated PM10 exposures could be associated with increased risk of CL in South Koreans. Further epidemiological and experimental studies are necessary to clarify the impact of chronic PM_10_ exposure on CL.

## Introduction

Laryngitis is a general term that describes inflammation of the larynx regardless of cause [1]. It is commonly classified into acute and chronic types. Acute laryngitis is most often caused by viral illnesses through direct inflammation of the vocal cords or from irritation due to postnasal drainage. Bacterial infections, such as acute epiglottitis, can also cause dysphonia but typically have other systemic symptoms as well as respiratory distress. Laryngitis is generally chronic when it lasts more than a few weeks and/or is caused by a mechanism that tends to have a longer course and require treatment. It is most commonly caused by laryngopharyngeal reflux, but other causes include poor laryngeal hygiene (smoking, excess alcohol, or caffeine intake), some bacterial and fungal infections (e.g., blastomycosis and tuberculosis), and more obscure conditions, such as the chronic laryngitis (CL) seen in glass blowers [2–4].

Air pollution is a heterogeneous mixture of gases, liquids, and solid particles, which all may be hazardous to health. Its main constituents, along with particulate matter (PM), are nitric oxides, sulfur oxides (SO_2_), carbon monoxide (CO), and ozone (O_3_). However, many epidemiologic studies have shown that PM exposure in particular is a risk factor for cardiorespiratory diseases, stroke, type 2 diabetes mellitus, and lung cancer [5,6]. Inhalable PM is classified according to the aerodynamic diameter of its particles: coarse (2.5–10 μm), fine (0.1–2.5 μm), and ultrafine (<0.1 μm). It is assumed that coarse PM is cleared in the upper airways, whereas fine PM reaches the lungs. Ambient air PM levels can be associated with increases in pneumonia and viral respiratory illness [7,8]. The proposed pathophysiological effects of PM are widely varied, including oxidative stress, mitochondrial perturbation, inflammation, protein denaturation, nuclear uptake, neuronal tissue uptake, phagocytic function perturbation, endothelial dysfunction, neo-antigen generation, and DNA damage [9].

Nationwide epidemiological studies conducted by government organizations can provide powerful data for investigating the national prevalence of disease conditions. The Korea National Health and Nutrition Examination Survey (KNHANES) was started in 1998 to examine the general health and nutrition status of populations in South Korea. From 2008 to 2012, 10,000–12,000 individuals were selected annually, and the participating household members were interviewed on their health and nutrition and asked to undergo a basic health assessment that included blood pressure measurements, blood and urine collection, a pulmonary function test, a dental examination, an ophthalmologic examination, and an otolaryngologic examination. The present study was undertaken to determine the national prevalence of CL in South Korea based on survey data obtained from the 2008 to 2012 KNHANES and to investigate associated factors. Furthermore, we investigated the effect of ambient particulate matter with an aerodynamic diameter less than 10 μm (PM_10_) for CL using large representative samples from KNHANES.

## Materials and Methods

### Ethics Statement

Written informed consent was obtained from all participants prior to the survey, and approval for this study was obtained from the Institutional Review Board of the Catholic University of Korea in Seoul, Korea.

### Study Population

This study was based on data from the KNHANES. The KNHANES is an ongoing population-based, cross-sectional, and nationally representative survey conducted by the Division of Chronic Disease Surveillance, Korean Center for Disease Control and Prevention. A field survey team that included an otolaryngologist, an ophthalmologist, and nurse examiners for health assessments moved with a mobile examination unit and performed interviews and physical examinations. Every year, 10,000–12,000 individuals in about 4,600 households are selected from a panel to represent the population by using a multistage clustered and stratified random sampling method that is based on the National Census Data. The survey consists of a health interview, a nutritional survey, and a health examination survey. The survey amasses data via household interviews and by direct standardized physical examinations conducted in specially equipped mobile examination centers. The KNHANES methodology has been described in detail previously [10,11]. A total of 4,000 households in 200 enumeration districts are selected annually by a panel that represents the civilian, noninstitutionalized South Korean population. All members of each selected household were asked to participate in the survey. The participation rate between 2008 and 2012 ranged from 77.8 to 82.8%. The KNHANES yields two main sampling weights: one for the persons who complete detailed interviews and the other for the subset who complete clinical evaluations.

### Survey for chronic laryngitis

Participants who were ≥ 19 years of age were examined. A laryngeal examination was performed using a 4 mm 70° angled rigid endoscope with a CCD camera. The Epidemiologic Survey Committee of the Korean Otolaryngologic Society prepared a CL decision protocol. Laryngoscopic findings of laryngitis and/or inflammation, including Reinke’s edema, pseudosulcus, erythema, edema, or thick endolaryngeal mucus were diagnosed as CL. The Epidemiologic Survey Committee of the Korean Otolaryngologic Society verified the quality control of the survey, which was conducted by periodically visiting the mobile examination units, periodic education of the participating residents, obtaining the laryngeal examination data, and data proofing using video documentation of the larynx throughout the study. Then, the two otolaryngologic surgeons from the Korean Otolaryngologic Society verified the video documentation and assessed the disease decision protocol. Documentation of the video was obtained as 640 × 480-sized Audio Video Interleave files, which were compressed by DivX 4.12 codec using a compression rate of 6 Mb/sec.

### Air pollutants

The air pollutants considered in this study included SO_2_, PM_10_, O_3_, nitrogen dioxide (NO_2_), and CO. Data were available from 283 monitoring stations in South Korea from January 2008 to December 2012. The average daily and annual concentrations from these stations were obtained from the Ministry of Environment. South Korea is divided into 16 administrative divisions including nine provinces (Gyeonggi, Gangwon, Chungbuk, Chungnam, Jeonbuk, Jeonnam, Gyeongbuk, Gyeongnam, and Jeju), six metropolitan cities (Busan, Daegu, Incheon, Gwangju, Daejeon, and Ulsan), and one capital metropolitan city (Seoul). Using average concentrations of air pollutants across 283 sites, we calculated average annual concentrations for the 16 administrative divisions of South Korea. The methods of measurement used for each air pollutant were the beta-ray absorption method (PM_10_), the pulse ultraviolet fluorescence method (SO_2_), the chemiluminescent method (NO_2_), the ultraviolet photometric method (O_3_), and the non-dispersive infrared method (CO).

### Statistical analysis

Statistical analyses were performed using the SAS survey procedure (ver. 9.3; SAS Institute, Cary, NC, USA) to reflect the complex sampling design and sampling weights of KNHANES and to provide nationally representative prevalence estimates. The procedures included unequal probabilities of selection, oversampling, and nonresponse so that inferences could be made about the Korean adolescent participants.

The prevalence and 95% confidence intervals (CIs) for CL were calculated. In the univariate analysis, the Rao-Scott chi-square test (using PROC SURVEYFREQ in SAS) was used to test the association between CL and risk factors in a complex sampling design. Participants’ characteristics were described using means and standard errors for continuous variables and numbers and percentages for categorical variables. Multiple logistic regression analyses were used to examine the association between CL and PM_10_. First, we adjusted for age and gender (Model 1). Then, we adjusted for age, gender, and other confounders that showed borderline significant differences f (P < 0.150) (Model 2). P-values were two-tailed and a P < 0.05 was considered significant.

## Results

### Prevalence of chronic laryngitis

**Fig 1:**
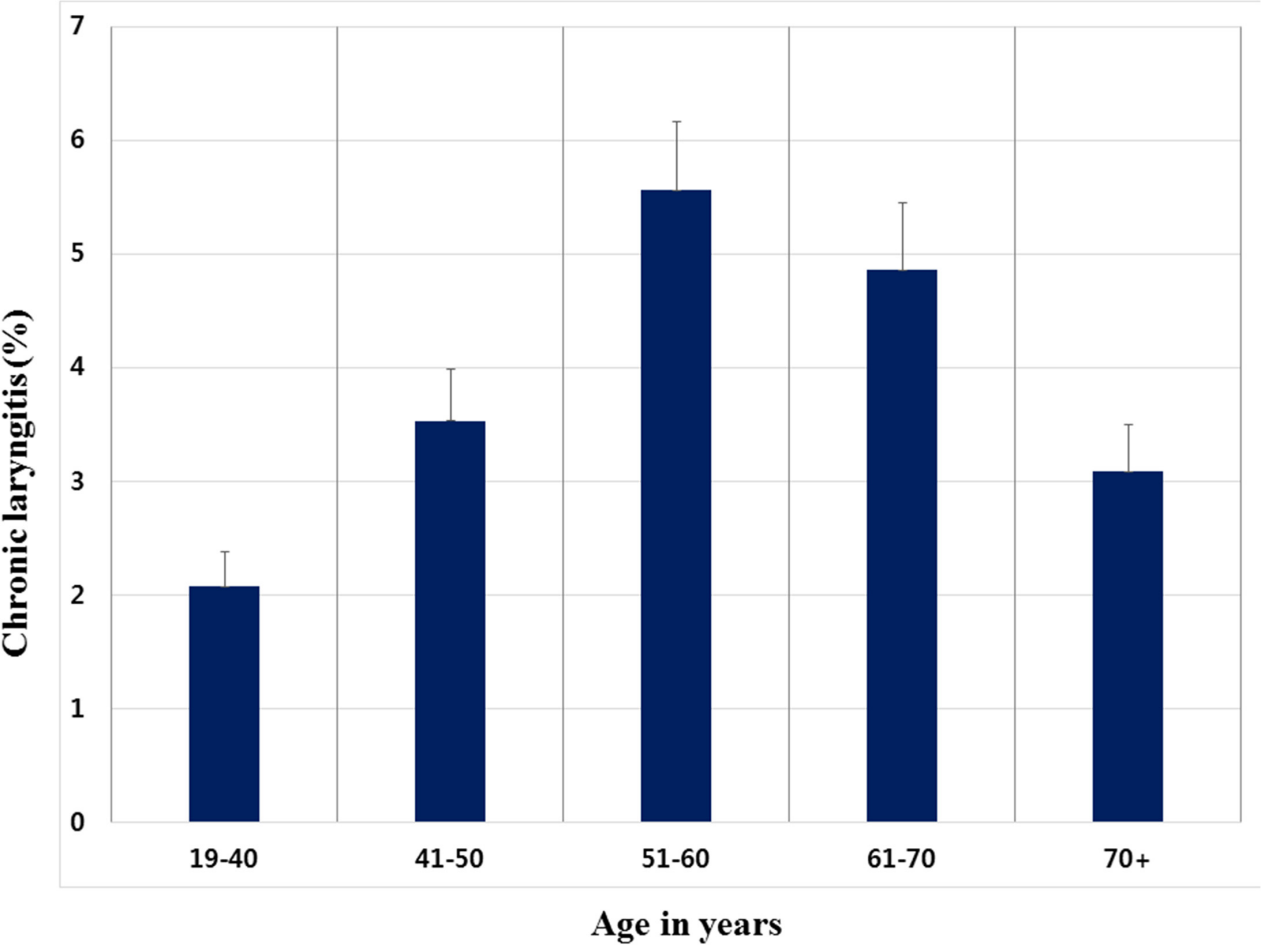
Overall chronic laryngitis prevalence by age group.

Among the 21,116 participants ≥ 19 years of age, 740 had experienced CL; the weighted prevalence of CL was 3.37 ± 0.30% (95% CI, 2.79–3.95%). The baseline characteristics of the study subjects according to CL are shown in Table 1. The mean age of those with CL was significantly higher than those without CL (P < 0.0001), and the prevalence of CL was higher in men than in women (P < 0.0001). Among variables regarding health behavior patterns, current smoking was significantly associated with CL (P < 0.0001). Those with CL had a lower household income (P = 0.0068). CL tended to be most prevalent among individuals in their fifties (5.56 ± 0.60%) and sixties (4.86 ± 0.59%) (Fig 1).

**Table 1:** Analysis of factors potentially associated with chronic laryngitis (n = 21116).

Parameter		N	Chronic Laryngitis		
			Yes (n = 740)	No (n = 20376)	P-value
**Age (years)** ^**a**^			49.2 (0.8)	44.5 (0.3)	<0.0001*
**Gender (%)** ^**b**^					<0.0001*
	**Male**	9105	62.0 (2.6)	49.4 (0.5)	
	**Female**	12011	38.0 (2.6)	50.6 (0.5)	
**Smoking status (%)** ^**b**^					<0.0001*
	**Current smoker**	4503	35.8 (2.9)	26.4 (0.5)	
	**Ex-/No-smoker**	16383	64.2 (2.9)	73.6 (0.5)	
**Alcohol consumption (%)** ^**b**^					0.1942
	**Frequently**	1775	12.4 (1.7)	10.3 (0.4)	
	**Occasionally/Rarely**	19341	87.6 (1.7)	89.7 (0.4)	
**Routine exercise (%)** ^**b**^					0.4173
	**Yes**	4683	24.2 (2.1)	22.5 (0.5)	
	**No**	16159	75.8 (2.1)	77.5 (0.5)	
**Residential area (%)** ^**b**^					0.4046
	**Urban**	16114	83.0 (3.4)	80.0 (1.7)	
	**Rural**	5002	17.0 (3.4)	20.0 (1.7)	
**Income (%)** ^**b**^					0.0051*
	**Lower**	4241	20.9 (2.2)	15.7 (0.6)	
	**Lower middle**	5265	27.0 (2.1)	26.7 (0.7)	
	**Upper middle**	6530	26.3 (2.5)	29.4 (0.7)	
	**Upper**	6261	25.8 (2.4)	28.1 (0.8)	
**Education level (%)** ^**b**^					0.0001*
	**University or higher**	5833	22.2 (2.0)	31.1 (0.8)	
	**High school**	7158	41.3 (2.4)	39.8 (0.7)	
	**Middle school**	2301	11.2 (1.3)	10.2 (0.3)	
	**Elementary school**	5554	25.3 (2.3)	18.9 (0.6)	
**Waist circumference (cm)** ^**a**^			83.6 (0.5)	80.8 (0.1)	<0.0001*
**Body mass index (kg/m** ^**2**^ **)** ^**a**^			24.3 (0.2)	23.6 (0.0)	<0.0001*
**PM 10 (㎍/m** ^**3**^ **)** ^**a**^			51.7 (0.5)	50.7 (0.2)	0.0621
**CO (ppm)** ^**a**^			0.55 (0.01)	0.54 (0.000)	0.5579
**NO**_**2**_ **(ppm)** ^**a**^			0.0262 (0.001)	0.0255 (0.000)	0.2943
**O**_**3**_ **(ppm)** ^**a**^			0.0229 (0.000)	0.0233 (0.000)	0.1182
**SO**_**2**_ **(ppm)** ^**a**^			0.0054 (0.000)	0.0054 (0.000)	0.2938

^a^Continuous variables are denoted by mean (SE)

^b^Values are percent (SE).

* Significant at p<0.05”

### PM_10_ and chronic laryngitis

**Fig 2:**
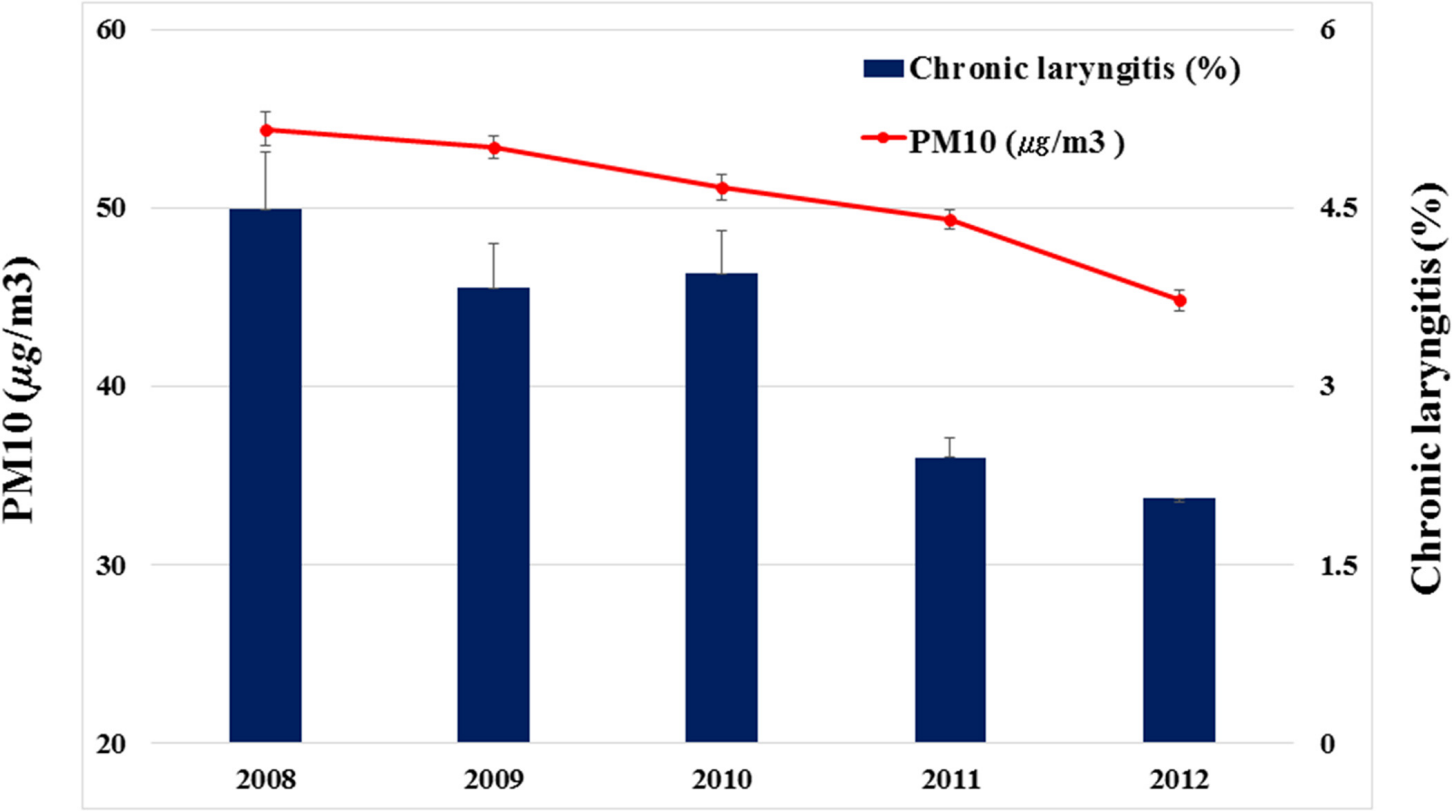
Trends in chronic laryngitis and ambient particulate matter with an aerodynamic diameter less than 10 μm (PM_10_) in 2008–2012, Korea.

A significant decrease over time was observed in the annual average concentrations of PM_10_ for the 16 administrative divisions of South Korea (54.39 μg/m^3^ for 2008, 51.17 μg/m^3^ for 2010, and 44.81 μg/m^3^ for 2012; P for trend < 0.0001) and the prevalence of CL (4.48% for 2008, 3.95% for 2010, and 2.06% for 2012; P for trend = 0.0049) (Fig 2). The annual prevalence of CL were associated with annual average concentrations of PM_10_ with borderline significance (P = 0.0621). However, no correlation was detected between prevalence of CL and SO_2_ (P = 0.2938), O_3_ (P = 0.1182), NO_2_ (P = 0.2943), or CO (P = 0.5579).

Table 2 shows the relationship between PM_10_ and CL using logistic regression models.

**Table 2:** Logistic regression models of PM_10_ for chronic laryngitis (n = 21116).

Parameter	Model 1			Model 2		
	OR	95% CI	P-value	OR	95% CI	P-value
**Age**	1.020	1.013–1.026	<0.0001*	1.020	1.012–1.029	<0.0001*
**Gender**	0.573	0.460–0.712	<0.0001*	0.734	0.568–0.948	0.0179*
**PM**_**10**_ **(per 10 ㎍/m** ^**3**^ **) Smoking status**	1.372	1.008–1.867	0.0445*	1.378	1.006–1.886	0.0457*
**Income**				1.483	1.123–1.958	0.0054*
**Education level**				1.109	0.837–1.470	0.4696
**Waist circumference**				1.081	0.805–1.453	0.6036
**Age**				1.018	1.007–1.029	0.0016*

OR = odds ratio; CI = confidence interval

* Significant at p<0.05

When age, sex, PM_10_, smoking status, income, education level, waist circumference were included in a regression model, PM_10_ (OR, 1.378; 95% CI, 1.006–1.886, P = 0.0457), age (OR, 1.02; 95% CI, 1.012–1.029, P < 0.0001), sex (OR, 0.734; 95% CI, 0.568–0.948, P = 0.0179), smoking status (OR, 1.483; 95% CI, 1.123–1.958, P = 0.0054), and waist circumference (OR, 1.018; 95% CI, 1.007–1.029, P = 0.0016) were significantly associated factors (Model 2). Income and education level did not show a significant correlation with CL.

## Discussion

This is the first epidemiological study to investigate the prevalence and associated factors of CL based on representative data from a government-centered survey in South Korea. Accurate epidemiological information may contribute to the proper provision of healthcare, preventive screenings, and rehabilitative services.

The term laryngitis generically refers to inflammation of the larynx tissues. CL usually develops gradually, and the underlying signs and symptoms can wax and wane over very long periods of time; some granulomatous forms can result from a single traumatic insult, and others may emerge when the larynx is repetitively exposed to the offending agent over a longer duration. Laryngitis is considered a common condition, and primary care practitioners and otolaryngologists see it often. However, the incidence of laryngitis has never been well defined [1]. Coyle et al. reported that the prevalence of laryngitis was 8.5% in 1,158 new patients seen by participating otolaryngologists [12]. Daniel et al. reported that a yearly incidence of CL was 3.47 cases per 1,000 people [13]. Signs and symptoms of laryngitis that last weeks or months and that may vary in severity over this period of time, are rarely caused by infectious or mechanical pathophysiological processes. Rather, patients who present with histories of persistent or episodic sore throats, globus sensations, hoarse voices, odynophagia, and coughing spells most often are suffering from the adverse effects of chronic irritative or contact laryngitis. Laryngeal examinations almost always reveal diffuse but variably severe supraglottal and glottal erythema and edema. Excessive and sticky-thick mucus secretions may be observed in the valleculae, piriform sinuses, and endolarynx. There are many potentially offending agents that are causally related to these pathological sequelae: (1) gastric acids that reflux into the laryngopharynx, (2) inhaled substances such as tobacco and industrial smoke, and (3) inhalation or ingestion of noxious or toxic chemicals [14]. In this study, the prevalence of CL was 3.37%. A significant relationship was observed between CL and older age, male gender, current smoker, and obesity. Age can be affective on function of esophageal function and CL. Oropharyngeal function has been changed with advancing years. By the changes that reduced the driving force of the tongue, decreased amplitude of pharyngeal wall contraction, and reduced pharyngeal swallowing, food retention can be located in the valleculae and piriform sinuses [15]. The afferent arm of laryngo-upper esophageal sphincter reflex is impaired, and the gag reflex is reportedly absent in 40% of the healthy elderly [15]. Also, there were interesting findings of gender based difference. Smoking may be the cause of gender based difference for CL. Smoking weakened the pharyngoglottal closure reflex, pharyngo-upper esophageal contractile reflex and reflexive pharyngeal swallow [16]. Korea is a rapidly westernizing country from a traditional Asian cultural background. According the KNHANES, cigarette smoking prevalence in men has fallen from 66% in 1998 to 47% in 2009. Prevalence of cigarette smoking in women stayed between 5.3 to 7.4% during the same period. National Smoking Prevalence Survey conducted by Korean Association of Smoking and Health provided earlier prevalence data, which reported adult cigarette smoking prevalence of 79.3% in men and 12.6% in women [17]. The prevalence has been decreased to 39.0% in men and 1.8% in women by 2011 [17].

Ambient PM is a ubiquitous atmospheric aerosol with both anthropogenic and natural sources that has been associated with various health effects [18]. PM deposits in the nose, pharynx, larynx, trachea, bronchi, and distal lung and, accordingly, the respiratory tract is the system most frequently infected after such exposure [19]. Coarse PM, with an aerodynamic diameter of 2.5–10 μm, deposits mainly in the head and large conducting airways. PM exposure can increase the risk for infection by altering various defense mechanisms present in the respiratory tract. Pathways of microbial clearance from the lung are decreased by particles; some portion of this effect is attributable to (1) impaired mucociliary function and (2) diminished phagocytosis by macrophages [20,21]. PM frequently contains various immunogenic substances, such as fungal spores and pollen, which have been independently associated with exacerbation of asthma symptoms [22, 23]. Experimental exposure to PM results in oxidative stress, airway hyper-responsiveness, and airway remodeling, either alone or in combination with allergic sensitization [24]. There appears to be a dose-response relationship between PM and risk for infections. Exposures with the greatest particle burden are associated with the largest increases in the incidence and/or prevalence of infectious disease [25]. In this cohort, the prevalence of CL was associated with annual average concentrations of PM_10_ with borderline significance. Furthermore the prevalence rate of CL showed a decreased trend with decreasing annual average concentrations of PM_10._ CL was positively associated with PM_10_ (OR, 1.378) as well as smoking status (OR, 1.483). Possible explanations for this may include that cigarette smoking is the particle-related exposure that presents the greatest PM burden, and smokers have more respiratory tract infections than their non-smoking peers [26–28].

In our study, PM_10_ concentration was the only air pollutant associated with CL in the multivariate logistic regression analyses. Fine and ultrafine dust has recently become a major public concern in Korea. Korea is affected by massive quantities of airborne dust particles caused by sandstorms and anthropogenic pollutant particles. Yellow dust (also called yellow sand or Asian dust), which originates from the deserts of Mongolia, northern China, has been an issue for decades in Korea. The major anthropogenic source of the dust is combustion products of fossil fuel. The maximum PM_10_ concentration observed in Korea frequently exceeds 1000 μg/m^3^ during the dust event period especially in spring [29]. Though further investigation is required for the association between CL and air pollutants, our results indicate that chronic exposure to disturbing environment can be a potential risk factor of CL.

There were a few limitations in this study. First, we do not know the relative severity or grade of the CL in the absence of objective testing and more detailed questions. Second, the present study was cross-sectional. We were unable to analyze the temporal association between CL and air pollutants. Adequate assessment of environmental exposures that vary within communities in population-based epidemiologic studies is limited by the expense involved in obtaining measurements at multiple locations, often for prolonged periods. Thus, our study is unable to examine within-city variability in air pollutant concentrations, which can be as large or larger than between-city variability. Although the causal relationship of risk factors with CL is inconclusive, the results may be reliable because this is a population-based study all around the country. Third, there may be some response bias on reporting of several parameters such as smoking, alcohol habits, and income, because the KNHANES was conducted by using self-administered questionnaires. Finally, the chronological decrease in the annual average concentrations of PM_10_ may have not been sufficient to cause a chronological decrease in the rate of CL in this study. Reflux esophagitis and drug history may have some role in decreasing the incidence of laryngopharyngeal reflux in our study subjects. Although we examined chronological changes in the incidence of CL, the rate of reflux esophagitis and precise drug history were not assessed. Future studies employing prospective, randomized methods to examine the CL are warranted.

In conclusion, our study is significant as an analysis of predictive factors associated with CL and PM_10_ on a nationally representative scale. Public acknowledgment and further intervention to modify factors associated with CL are required for preventing and managing the condition.
